# Hemangioblastoma Instead of Renal Cell Carcinoma Plays a Major Role in the Unfavorable Overall Survival of Von Hippel-Lindau Disease Patients

**DOI:** 10.3389/fonc.2019.01037

**Published:** 2019-10-09

**Authors:** Bowen Zhou, Jiangyi Wang, Shengjie Liu, Xiang Peng, Baoan Hong, Jingcheng Zhou, Kaifang Ma, Jiufeng Zhang, Lin Cai, Kan Gong

**Affiliations:** ^1^Department of Urology, Peking University First Hospital, Beijing, China; ^2^Institute of Urology, Peking University, Beijing, China; ^3^National Urological Cancer Center, Beijing, China; ^4^Department of Urology, Beijing Hospital, Beijing, China; ^5^Department of Urology, Fudan University Shanghai Cancer Center, Shanghai, China; ^6^Department of Urology, The Second Affiliated Hospital of NanChang University, Jiangxi, China

**Keywords:** von Hippel-Lindau disease, risk factor, genetic cancer syndrome, prognosis, survival

## Abstract

Von Hippel-Lindau (VHL) disease is a hereditary cancer syndrome characterized by poor survival. The effect of the involvement of each organ on survival remains unclear. Our study aimed to study the effect of the involvement of each organ on survival in VHL disease patients. We retrospectively analyzed 336 patients from 125 families. The onset age was compared between different groups using Mann-Whitney U test and Kruskal-Wallis test. Univariate and multivariate time-dependent Cox regression analyses were conducted to evaluate how survival was influenced by the involvement of each organ. The median survival time for VHL disease patients was 66 years. The onset age was earlier in the central nervous system (CNS) group than in the abdominal group. The involvement of central nervous system hemangioblastoma (CHB) and retinal hemangioblastoma (RA) were independent risk factors for overall survival. The involvement of renal cell carcinoma (RCC) was not a significant risk factor for overall survival. Only RA was a risk factor for CHB-specific survival. This study analyzed the relationship between organ involvement and survival of VHL patients. This may help guide future genetic counseling and clinical decision-making.

## Introduction

Von Hippel-Lindau (VHL) disease (MIM 193300) is an autosomal dominant tumor syndrome caused by germline mutations in the tumor suppressor gene *VHL* located on 3p25. The incidence is ~1 in 36,000–40,000 live births and has high penetrance (1–3). Benign and malignant tumors including central nervous system hemangioblastoma (CHB), retinal hemangioblastoma (RA), renal cell carcinoma (RCC), pheochromocytoma (PHEO), pancreatic lesions, including pancreatic cystic lesion, and pancreatic neuroendocrine tumors (PCL/PNET), endolymphatic sac tumor (ELST) and papillary cystadenomas of the epididymis or broad ligament are the main manifestations of this disease (4–11). Both intra- and interfamilial phenotypic heterogeneity can be seen in VHL disease patients (12, 13).

Many studies indicated that the survival of VHL disease patients was worse than the general population. A study in 1990 showed that the median survival was 49 years (14). A recent study reported the estimated life expectancy for male and female patients was 67 and 60 years, respectively (15). In 2012, a study reported that the median life expectancy for VHL disease patients was 52 years (16). Previously reported risk factors for survival of VHL disease patients included early birth year, fewer monitoring visits, female gender, positive family history, early disease onset, truncating mutation type and RCC larger than 3 cm (15, 17, 18). However, no previous research has studied the effects of the involvement of each organ in a large patient group.

The effect of the involvement of different organs on survival have been studied in other systemic diseases. For example, uterine involvement indicates worse survival in patients with diffuse large B-cell lymphoma than in those with the involvement of other organs (19). Yuda et al. reported that pericardial effusion and multiple organ involvement were predictors of mortality in patients with systemic light chain amyloidosis (20). Although some previous studies established genotype-phenotype correlations in VHL disease: patients with missense mutations are more likely to develop pheochromocytoma, and patients with truncating mutations are more likely to develop RCC and CHB (21–23). The existing genotype-phenotype correlations can not explain other complex phenotypes. Hence it's vital to study the prognosis of VHL disease from an organ involvement perspective (23, 24).

In this study, we aimed to study the overall survival and CHB-specific survival in a large VHL disease patient group that includes asymptomatic mutation carriers and to investigate the influence of CHB, RCC, RA, PCL/PNET and PHEO, in order to improve genetic counseling and clinical treatment strategies for VHL disease patients.

## Materials and Methods

### Patients and Samples

We recruited all the patient information stored in the database of VHL disease patients at Peking University First Hospital. The diagnosis was established when the patient was found to carry a germline *VHL* gene mutation or fulfilled the previously described clinical criteria (25, 26). At least one patient was diagnosed by *VHL* mutation test in each family, except those refused the test. In total, 376 patients from 134 families were diagnosed. Clinical data including sex, family history, mutation type, onset age of each organ and the cause of death were obtained by reviewing medical records or interviewing family members. Forty patients were excluded from the study due to unclear data. A total of 336 patients from 125 families were finally enrolled in this cohort, in which 298 of the patients had at least one VHL disease-related lesion and 38 patients were asymptomatic mutation carriers.

We recorded the survival status of the 336 patients from birth to death or to the end of follow-up in June 2017. The median follow-up time was 37 years/person (range 1–75 years), with a total of 12,966 person-years.

### Genetic Analysis

A QIAamp DNA Blood Mini Kit (QIAGEN, Germany) was used to extract genomic DNA from peripheral blood of suspected patients. Three coding exons and their flanking intronic regions were amplified by PCR using primers described previously (27). Missense mutations, splicing mutations, and small indels were detected by direct sequencing. A multiplex ligation-dependent probe amplification kit (MLPA, P016-C2, MRC-Holland, Amsterdam) was used to detect large deletions. Large exon deletions were confirmed by real-time quantitative PCR with the primers described by Ebenazer et al. (28). Patients were divided into a missense mutation group (*n* = 162) and a truncating mutation group (*n* = 174) based on the criteria described previously (18).

### Statistical Analysis

Patient demographics and basic clinical data were analyzed using descriptive statistics. The onset age of lesions in different organs was compared using Kruskal-Wallis test; pairwise comparisons were conducted, and the *P* value was determined after Bonferroni adjustment. The onset age in the CNS group and abdominal group was compared using Mann-Whitney U test.

Because follow-up was performed from birth, patients before disease onset and after disease onset have different risks for survival. This situation does not fit the requirements of the proportional hazard model, making Cox regression analysis inappropriate. Instead, the involvement of every organ was treated as a time-dependent covariate in the Cox regression model. Univariate and multivariate Cox regression analyses were used to assess the effects of CHB, RCC, RA, PCL/PNET, and PHEO on overall survival. We then used univariate and multivariate Cox regression analyses to determine the effect of RCC, RA, PCL/PNET, and PHEO on CHB-specific survival because CHB is the most common cause of death in VHL disease patients.

Statistical analysis was performed using SPSS 22.0 and GraphPad Prism software (version 6). *P* < 0.05 was considered statistically significant. For pairwise comparisons, Bonferroni's adjustment was conducted.

### Ethics Statement

This study was approved by the Institutional Review Board of Peking University First Hospital (Beijing, China), and written consent was obtained from all patients.

## Results

### Clinical Characteristics of VHL Disease Patients

**Figure 1:**
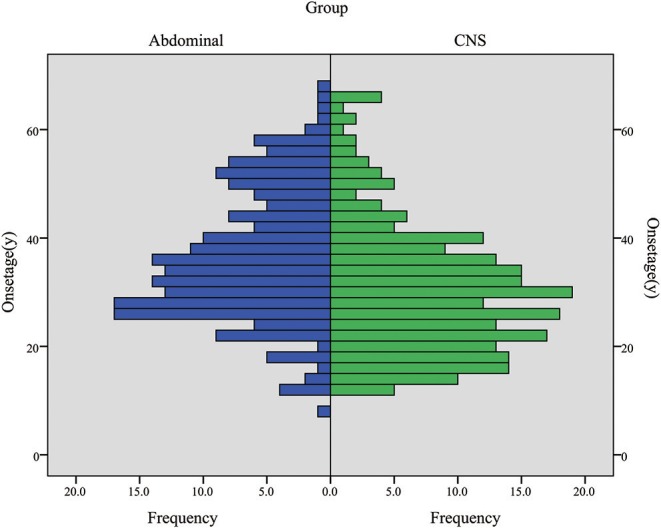
The onset age frequency in CNS group (including CHB & RA patients) and abdominal group (including RCC, PHEO & PCL/PNET patients).

CHB was the most common lesion in our study, and more than half of the patients had CHB lesions (62.2%). PCL/PNET was the second most common lesion (46.4%). Nearly two-thirds of the deceased patients died of CHB (66.2%). The prevalence of CHB and RCC in this study was generally in line with the results of previous studies (Supplementary Table 1). The median years of age were ≤ 30 years at the onset of CHB and RA, and were >30 years at the onset of RCC, PHEO and PCL/PNET (Table 1). We combined CHB and RA into a central nervous system (CNS) group, and RCC, PHEO and PCL/PNET into an abdominal group. The distribution of the onset age in the two groups is shown in Figure 1. The median years of age at the onset of tumors in abdominal and CNS groups were 34 and 29 years, respectively. Mann-Whitney *U* test indicated that the onset age was significantly younger in CNS group than in abdominal group (*P* < 0.001).

**Table 1:** Age at the onset of VHL-related lesions.

**Lesion**	**Mean ± SD**	**Median (range)**
CHB	31.4 ± 11.0	30 (12–66)
RCC	38.2 ± 11.3	36.5 (18–69)
RA	30.3 ± 13.3	28.5 (11–66)
PCL/PNET	36.3 ± 11.9	34 (12–67)
PHEO	35.0 ± 13.6	36 (8–62)

### Overall Survival and CHB-Specific Survival in VHL Disease Patients

**Figure 2:**
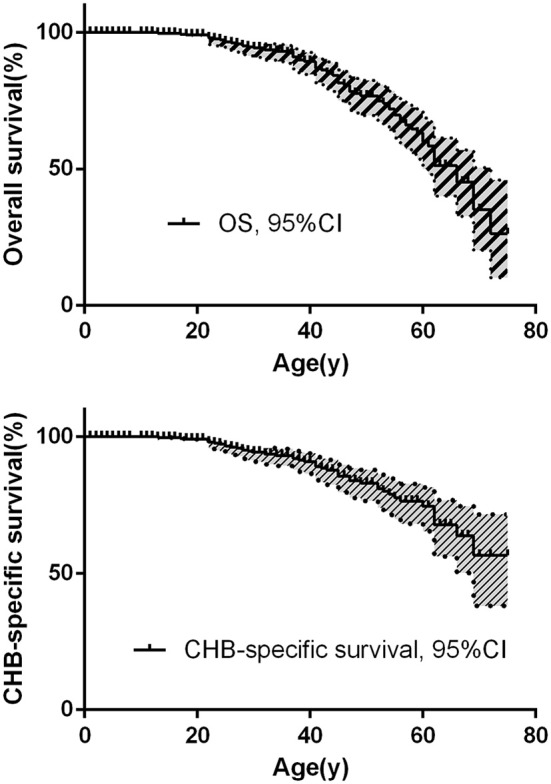
**(A)** The overall survival of VHL patients. **(B)** CHB-specific survival of VHL patients.

The overall survival and CHB-specific survival are shown in Figures 2A,B. The median survival time for VHL disease patients was 66 years. Together, CHB and RCC accounted for 95.6% of all death cases. Nearly two thirds of the patients died of CHB-related conditions and more than one-third of the patients died of RCC. Among 209 patients with CHB, 45 patients died of CHB, 10 patients died of RCC. Among 138 patients with RCC, 16 patients died of RCC, 4 patients died of CHB. Among 86 patients with both lesions, CHB and RCC contributed equally to deceased patients, both cause 8 deaths. CHB was the more common cause of death than RCC in our study (Table 2).

**Table 2:** Baseline clinical characteristics of VHL disease patients.

	**Patient (*n*)**		**Patient (*n*)**
Overall	336	CHB involvement	
Sex		Yes	209 (62.2%)
Male	160 (47.6%)	No	127 (37.8%)
Female	176 (52.4%)	RCC involvement	
Family history		Yes	138 (41.1%)
Yes	265 (78.9%)	No	198 (58.9%)
No	71 (21.1%)	RA involvement	
Mutation type		Yes	80 (23.8%)
Missense	162 (48.2%)	No	256 (76.2%)
Truncating	174 (51.8%)	PCL/PNET involvement	
First onset organ		Yes	156 (46.4%)
CHB	157 (46.7%)	No	180 (53.6%)
RCC	52 (15.5%)	PHEO involvement	
RA	37 (11.0%)	Yes	46 (13.7%)
PCL/PNET	16 (4.8%)	No	290 (86.3%)
PHEO	19 (5.7%)	Total number of death	68
Two or more organs^*^	17 (5.1%)	CHB	45 (66.2%)
Mutation carriers	38(11.3%)	RCC	20 (29.4%)
		PHEO	2 (2.9%)
		Unrelated to VHL	1 (1.5%)

* *Two or more organs involved at disease onset*.

### Univariate and Multivariate Cox Regression Analyses for Overall Survival

In univariate analysis, CHB (HR 2.583, 95% CI 1.545–4.318, *P* = 0.001) and RA (HR 2.832, 95% CI 1.581–5.071, *P* = 0.001) were associated with poorer overall survival. RCC did not significantly affect the overall survival (HR 1.533, 95% CI 0.835–2.814, *P* = 0.168). Multivariate Cox regression analysis showed similar results. CHB (HR 2.426, 95% CI 1.433–4.106, *P* = 0.001) and RA (HR 2.440, 95% CI 1.337–4.455, *P* = 0.004) were predictors of poorer overall survival. Again, the effect of RCC on overall survival was not statistically significant (HR 1.554, 95% CI 0.747–3.233, *P* = 0.239; Table 3).

**Table 3:** Univariate and multivariate Cox regression analyses for overall survival.

**Involvement**	**Univariate analysis**		**Multivariate analysis**	
	**HR (95% CI)**	***P* value**	**HR (95% CI)**	***P* value**
CHB (Yes vs. No)	2.583 (1.545–4.318)	**0.001**	2.426 (1.433–4.106)	**0.001**
RCC (Yes vs. No)	1.533 (0.835–2.814)	0.168	1.554 (0.747–3.233)	0.239
RA (Yes vs. No)	2.832 (1.581–5.071)	**0.001**	2.440 (1.337–4.455)	**0.004**
PCL/PNET (Yes vs. No)	0.954 (0.476–1.913)	0.894	0.534 (0.232–1.228)	0.140
PHEO (Yes vs. No)	0.586 (0.182–1.886)	0.370	0.814 (0.250–2.649)	0.733

*HR, hazard ratio; CI, confidence interval. Y, yes. N, no*.

*Bold values are statistically significant (P < 0.05). The involvement of each organ was treated as a time-dependent covariate*.

### Effects of Different Organ Involvement on CHB-Specific Survival

Because CHB accounted for nearly two-thirds of the death (66.2%, 45 of 68) and CHB was a significant risk factor for overall survival in both univariate and multivariate Cox regression analyses, we further studied the effect of other organ involvement on CHB-specific survival. The involvement of every organ was treated as a time-dependent covariate.

In univariate Cox regression analysis, only RA was a risk factor for CHB-specific survival (HR 2.080, 95% CI 0.962–4.495) but the *P* value was not significant (*P* = 0.063). In the multivariate analysis, RA was a significant risk factor for CHB-specific survival (HR 2.210, 95% CI 1.005–4.862, *P* = 0.049; Table 4).

**Table 4:** Univariate and multivariate time-dependent Cox regression analyses of the effect of different organ involvement on CHB-specific survival.

**Involvement**	**Univariate analysis**		**Multivariate analysis**	
	**HR (95%CI)**	***P* value**	**HR (95%CI)**	***P* value**
RCC (Y vs. N)	0.576 (0.200–1.661)	0.308	0.523 (0.152–1.797)	0.303
RA (Y vs. N)	2.080 (0.962–4.495)	0.063	2.210 (1.005–4.862)	**0.049**
PCL/PNET (Y vs. N)	0.769 (0.294–2.010)	0.592	1.003 (0.334–3.015)	0.995
PHEO (Y vs. N)	0.043 (0.001–9.138)	0.250	0.001 (0.001–9.458)	0.974

*HR, hazard ratio; CI, confidence interval. Y, yes. N, no*.

*Bold values are statistically significant (P < 0.05)*.

*The involvement of each organ was treated as a time-dependent covariate*.

### The Effect of RCC Size on Patient Survival

Our database contained only 51 patients with a complete natural history of RCC. We further categorized these patients into 2 groups: the first group, RCC diameter of <3 cm throughout their natural history (*n* = 15), and the second group, RCC diameter of >3 cm in the period of their natural history (*n* = 36). All the patients in the first group survived, Three patients in the second group died by the end of follow-up; one died of a CHB-related condition, and the other two died of metastatic RCC with the RCC diameters of 7 and 8.5 cm at the time of diagnosis.

## Discussion

In this study, CHB and RCC were still the leading causes of deaths in VHL patients, together caused 95.6% of all deaths. We analyzed the effect of every involved organ on overall survival and CHB-specific survival of VHL disease patients. CHB and RA were independent risk factors for overall survival. Surprisingly, RCC was not a significant risk factor for overall survival. RA was the only risk factor for CHB-specific survival. In the past decades, the cause of death in VHL disease patients has shifted greatly. In studies on VHL disease published in the early years, CHB and RCC contributed approximately equally to the death of patients; in some studies, the number of death due to RCC was even twice more than that due to CHB (29). In recent studies, however, the proportion of CHB-related death was significantly higher than that of RCC-related death (Supplementary Table 1). Such discrepancy can partially explained by the development of advanced imaging technologies and treatment strategies including targeted therapy, minimally invasive surgery and active surveillance for RCCs, contributing to the early diagnosis and effective treatment before metastasis of RCCs. On the other hand, the pharmacological and surgical treatment for CHB are required to be further improved.

Although RCC in VHL disease is usually considered to be less malignant and less likely to metastasize and to have higher cancer-specific survival compared with sporadic clear cell RCC (30), RCC is still an important cause of death in VHL patients. Most deaths are due to tumor metastasis and tumor-related complications. Surprisingly in this study, the effect of RCC on overall survival was not significant. A Korean study including 24 VHL disease patients published in 2009 showed similar results (31). Previous studies have demonstrated that the RCC diameter of >3 cm is a risk factor for metastasis and overall survival (17, 32). In the VHL disease patient database we analyzed in this study, among the 51 patients with complete natural history of RCC tumor size, only 3 patients died during followup, rendering it impossible to perform survival analysis. Jilg et al. reported that the mean tumor diameter in patients without metastases was significantly smaller than that in patients with metastases (33). Duffey et al. reported in 2004 that patients with RCCs smaller than 3 cm were at a low risk of distant metastasis and recommended 3 cm as the cutoff value for surgical intervention (34). RCC is still an important cause of death in VHL disease patients. Tumor size and growth rate may be of greater significance for prognosis. Larger studies with longer follow-up periods are needed to study the relationship between survival and RCCs.

Although CHB is considered to be a benign tumor, it can cause severe neurological defects, such as hydrocephalus, herniation, and brain stem compression, and in rare cases intracranial hemorrhage (14, 29). Perioperative/postoperative complications can also lead to death in CHB patients. The frequency of CHB involvement in study sample may affect survival analysis. 62.2% (209 of 336) patients had CHB in our study, which is below the average number of patients with CHB in the studies listed in Supplementary Table 1. CHB accounted for 66.2% of all death patients in this cohort, which is higher than 6 of the 7 studies listed in Supplementary Table 1. This may be caused by birthyear of patients. In a Danish study published in 2016, CHB-related death rate rise from 42% (birth year 1901–1955, 18 of 50) to 83% (birth year 1956–2010, 5 of 6) as birth year increases, with a total CHB-related death rate of 51%. The author attributed such discrepancy to progress in diagnosis and treatment of RCC (15). However, in our study, 83.3% (280 of 336) patients were born after 1955, only 16.7% (56 of 336)patients were born before 1955. And CHB-related death rate was 60.1% for patient born before 1955, but increased to 70.1 and 75% for patients born during 1955–1975 and after 1975. RA causes blindness; however in our study, RA was a significant risk factor for overall survival and CHB-specific survival. Retina embryologically originates from brain, and both CHB and RA share the same histopathological structures (7). The oncogenesis of hemangioblastoma is related to the dysregulation of HIF pathway leading to the increase of vascular endothelial growth factor and erythropoietin (35, 36). Franke et al. reported that deletion mutations in *VHL* gene had a higher risk of CHB and RA (37). Thus, we hypothesize that RA may reflect a greater burden of CHB, leading to unfavorable survival of VHL disease patients.

Hemangioblastoma is a significant risk factor for survival. Another possible explanation for this is the earlier onset age of this tumor. Our previous research revealed that early onset age was a risk factor for overall survival and VHL disease-specific survival (18). The median age at the onset of CHB and RA were 28.5 and 30 years, respectively, being the earliest among all VHL-related lesions. When CHB and RA were combined as a CNS group, this group also had earlier onset age than abdominal group. We conducted a Kruskal-Wallis test to compare the onset age of the lesions in 5 organs, and *P* < 0.001 suggests a significant difference. We further made pairwise comparisons and the *P* value was valuated after Bonferroni adjustment, and significant differences of age onset were found between RA-PCL/PNET, RA-RCC, CHB-PCL/PNET, and CHB-RCC (Supplementary Table 2). Therefore, although hemangioblastoma is considered to be a benign tumor, its early onset may worsen the survival of the VHL disease patients.

Several previous studies that only included symptomatic patients may have biased results that overestimate the rate of involved organs and the effect of involved organs on patient survival (31, 38). We first used the time-dependent Cox regression analysis to evaluate the effect of every involved organ on the survival of VHL disease patients. In a previous study, overall survival was calculated from disease diagnosis to death; however asymptomatic mutation carriers can develop tumors later and do not fit the requirements of the proportional hazard model (17).

This large retrospective study focused on different organ involvement on survival in VHL disease patients from a clinical perspective. The life expectancy of VHL disease patients was 66 years. The presence of CHB and RA were the independent risk factors for overall survival. RCC was not a significant risk factor for overall survival. RA was a risk factor for CHB-specific survival. Patients with RA should have careful active surveillance due to the higher risk of death from CHB. Future larger studies with longer follow-up periods are needed to study the effect of RCC on survival of VHL patients. These findings may help guide future genetic counseling and clinical decision making.

## Data Availability Statement

The datasets generated for this study are available on request to the corresponding author

## Ethics Statement

The studies involving human participants were reviewed and approved by Institutional Review Board of Peking University First Hospital (Beijing, China). Written informed consent to participate in this study was provided by the participants' legal guardian/next of kin.

## Author Contributions

KG and LC were responsible for the concept and design of the study. BZ and JW dealt with the clinical data. SL and XP performed the statistical work. BH and JZho grafted the manuscript. KM and JZha provides the figures and tables. All authors revised the manuscript.

### Conflict of Interest

The authors declare that the research was conducted in the absence of any commercial or financial relationships that could be construed as a potential conflict of interest.

## Abbreviations

CHB: central nervous system hemangioblastoma
RA: retinal hemangioblastoma
RCC: renal cell carcinoma
PHEO: pheochromocytoma
PCL/PNET: pancreatic cystic lesion and pancreatic neuroendocrine tumors.
