# Supplementary material for: Hemangioblastoma Instead of Renal Cell Carcinoma Plays a Major Role in the Unfavorable Overall Survival of Von Hippel-Lindau Disease Patients
Source: Front Oncol. 2019 Oct 9;9:1037. doi: 10.3389/fonc.2019.01037 (PMC6794496; doi:10.3389/fonc.2019.01037)
Supplement: Supplementary file 1 [file Data_Sheet_1.docx]

Supplementary Material

**Supplementary Table 1. Frequency of organ involvement and cause of death in VHL disease patients in previous studies**

| **Publication year** | **Frequency of CHB;**  **frequency of RCC** |  | **Frequency of death cause** | **Reference** |
| --- | --- | --- | --- | --- |
| 1989 | 41.9% (18 of 43);  46.5% (20 of 43) |  | CHB: 21% (4 of 19)  RCC: 42% (8 of 19)  Other causes: 37% (7 of 19) | (Lamiell et al., 1989) |
| 1990 | 71.7% (109 of 152);  28.3% (43 of 152) |  | CHB: 41% (21 of 51)  RCC: 47% (24 of 51)  Other causes: 12% (6 of 51) | (Maher et al., 1990) |
| 1992 | 44.3%(43 of 97);  20 in 43 CHB-positive  patients (including with  renal cysts) |  | CHB: 82%(18 of 22)  Other causes: 18% (4 of 22) | (Neumann et al., 1992) |
| 1996 | ND*;  25.3% (21 of 83). |  | CHB: 47.7% (21 of 44)  RCC: 27.3% (12 of 44)  Pancreatic carcinoma: 6.8% (3 of 44)  PHEO: 4.5% (2 of 44)  Other causes: 13.6% (6 of 44) | (Maddock et al., 1996) |
| 1998 | 72% (155 of 215);  36.7% (79 of 215) |  | CHB: 61.5% (8 of 13)  RCC: 30.8% (4 of 13)  Other causes: 7.7% (1 of 13) | (Richard et al., 1998) |
| 1999 | 100% (14 of 14);  71.4% (10 of 14) |  | CHB: 27% (3 of 11)  RCC: 45% (5 of 11)  Pancreatic carcinoma: 9% (1 of 11)  Other causes: 18% (2 of 11) | (Niemela et al., 1999) |
| 2014 | 100% (225 of 225);  58.2% (131 of 225) |  | CHB: 25% (4 of 16)  RCC: 12.5% (2 of 16)  Other causes: 62.5% (10 of 16) | (Lonser et al., 2014) |
| 2018 | 62.2% (209 of 336);  41.1% (138 of 336) |  | CHB: 66.2% (45 of 68)  RCC: 29.4% (20 of 68)  PHEO: 2.9% (2 of 68)  Other causes:1.5% (1 of 68) | This study |

*Data from an unpublished French study included in this review. *ND, not detailed.

**Supplementary Table 2** Pairwise comparisons of age at the onset of VHL-related lesions in different organs

| Comparison groups | P value after Bonferroni adjustment |
| --- | --- |
| RA-CHB | 1.000 |
| RA-PHEO | 0.182 |
| RA-PCL/PNET | **0.002** |
| RA-RCC | **0.001** |
| CHB-PHEO | 0.358 |
| CHB-PCL/PNET | **0.001** |
| CHB-RCC | **0.001** |
| PHEO--PCL/PNET | 1.000 |
| PHEO-RCC | 1.000 |
| PCT-RCC | 1.000 |

Bold values are statistically significant (P<0.05)

Lamiell, J.M., Salazar, F.G., and Hsia, Y.E. (1989). von Hippel-Lindau disease affecting 43 members of a single kindred. *Medicine (Baltimore)* 68(1)**,** 1-29.

Lonser, R.R., Butman, J.A., Huntoon, K., Asthagiri, A.R., Wu, T., Bakhtian, K.D., et al. (2014). Prospective natural history study of central nervous system hemangioblastomas in von Hippel-Lindau disease. *J Neurosurg* 120(5)**,** 1055-1062. doi: 10.3171/2014.1.JNS131431.

Maddock, I.R., Moran, A., Maher, E.R., Teare, M.D., Norman, A., Payne, S.J., et al. (1996). A genetic register for von Hippel-Lindau disease. *J Med Genet* 33(2)**,** 120-127.

Maher, E.R., Yates, J.R., Harries, R., Benjamin, C., Harris, R., Moore, A.T., et al. (1990). Clinical features and natural history of von Hippel-Lindau disease. *Q J Med* 77(283)**,** 1151-1163.

Neumann, H.P., Eggert, H.R., Scheremet, R., Schumacher, M., Mohadjer, M., Wakhloo, A.K., et al. (1992). Central nervous system lesions in von Hippel-Lindau syndrome. *J Neurol Neurosurg Psychiatry* 55(10)**,** 898-901.

Niemela, M., Lemeta, S., Summanen, P., Bohling, T., Sainio, M., Kere, J., et al. (1999). Long-term prognosis of haemangioblastoma of the CNS: impact of von Hippel-Lindau disease. *Acta Neurochir (Wien)* 141(11)**,** 1147-1156.

Richard, S., Campello, C., Taillandier, L., Parker, F., and Resche, F. (1998). Haemangioblastoma of the central nervous system in von Hippel-Lindau disease. French VHL Study Group. *J Intern Med* 243(6)**,** 547-553.
